# Can Scat Analysis Describe the Feeding Habits of Big Cats? A Case Study with Jaguars (*Panthera onca*) in Southern Pantanal, Brazil

**DOI:** 10.1371/journal.pone.0151814

**Published:** 2016-03-22

**Authors:** Miriam L. L. Perilli, Fernando Lima, Flávio H. G. Rodrigues, Sandra M. C. Cavalcanti

**Affiliations:** 1 Programa de Pós-graduação em Ecologia, Departamento de Biologia Geral, Universidade Federal de Viçosa - UFV, Viçosa, Minas Gerais, Brazil; 2 Programa de Pós-graduação em Ecologia e Conservação, Universidade Federal do Mato Grosso do Sul - UFMS, Campo Grande, Mato Grosso do Sul, Brazil; 3 Instituto para Conservação dos Carnívoros Neotropicais - Pró-Carnívoros, Atibaia, São Paulo, Brazil; 4 IPÊ - Instituto de Pesquisas Ecológicas, Nazaré Paulista, São Paulo, Brazil; 5 Programa de Pós-graduação em Ecologia e Biodiversidade, Instituto de Biociências, Universidade Estadual Paulista – UNESP, Rio Claro, São Paulo, Brazil; 6 Departamento de Biologia Geral, Instituto de Ciências Biológicas, Universidade Federal de Minas Gerais - UFMG, Belo Horizonte, Minas Gerais, Brazil; University of Sydney, AUSTRALIA

## Abstract

Large cats feeding habits have been studied through two main methods: scat analysis and the carcasses of prey killed by monitored animals. From November 2001 to April 2004, we studied jaguar predation patterns using GPS telemetry location clusters on a cattle ranch in southern Pantanal. During this period, we recorded 431 carcasses of animals preyed upon by monitored jaguars. Concurrently, we collected 125 jaguar scats opportunistically. We compared the frequencies of prey found through each method. We also compared the prey communities using Bray-Curtis similarity coefficient. These comparisons allowed us to evaluate the use of scat analysis as a means to describe jaguar feeding habits. Both approaches identified prey communities with high similarity (Bray-Curtis coefficient > 70). According to either method, jaguars consume three main prey: cattle (*Bos taurus*), caiman (*Caiman yacare*) and peccaries (*Tayassu pecari* and *Pecari tajacu*). The two methods did not differ in the frequency of the three main prey over dry and wet seasons or years sampled. Our results show that scat analysis is effective and capable of describing jaguar feeding habits.

## Introduction

The ecological importance of large mammalian carnivores such as the jaguar (*Panthera onca*) is easily recognized, as even a few individuals can exert strong top-down control on prey populations and smaller predators [1–3]. These 'apex predators’ occupy an elevated position on the trophic ladder, and their role as ecosystem regulators is now firmly embedded in ecological theory [4–6]. The jaguar is the largest of the Neotropical cats and is considered Near Threatened by the IUCN [7], with a decreasing population trend. Habitat degradation, consequent loss of natural prey, and hunting pressure has led to a reduction in range and historical occurrence of the jaguar [8]. Because of these threats, jaguar populations are either restricted to remote areas with low human densities or forced to coexistence with man and livestock [9,10]. This coexistence increases the possibility of these carnivores preying on livestock instead of their natural prey [10–12]

The Pantanal in Brazil is a vast flood plain that shelters abundant wildlife and is considered essential for the long-term conservation of jaguars, as it holds the highest abundance of the species[8,13]. The region is characterized by large-scale private cattle ranches, and the extensive management of the cattle brings the herds into direct contact with jaguars, which eventually prey upon them [13–15]. Predation on livestock is considered the biggest source of conflict between jaguars and ranchers and is often used as a justification for jaguar poaching [9,12,15,16]. Studies on jaguar feeding habits are important not only to increase our knowledge on the foraging patterns of the species but also to provide subsidies to develop mitigation measures for livestock depredation.

The first studies on the feeding habits of jaguars emerged in the late 1970s and the 1980s in the Pantanal [13,17,18], Belize [19] and Peru [20]. We now know that the species feeds on a wide variety of prey, ranging from small rodents to large mammals such as the marsh-deer (*Blastocerus dichotomus*) and tapir (*Tapirus terrestris*). Much of the information currently available about jaguar diet originates from scats [14,19–38]. As a noninvasive method, scat analysis is an important tool for studying cryptic animals, which are difficult to observe and capture.

Another method of studying jaguar feeding habits is through the detection of kills by individuals monitored with telemetry [13–15]. With advances in global positioning system (GPS) telemetry technology for wildlife studies, it became possible to build more detailed databases on the feeding habits of large cats by detecting clusters of locations [15,39–41]. This method consists of investigating locations that have consecutive GPS records in close proximity to each other, indicating that the animal spent a considerable amount of time in a specific site [39]. These technologies are relatively recent and, despite generating high quality data, involve high costs and direct manipulation of individuals (*i*.*e*. physical constrain and anesthesia). A potential flaw in this method is that small and medium-sized species are often completely consumed or their remains can be carried out by scavengers, reducing the probability of detection and creating a bias for larger prey [42].

Here, we compare the results of two methods of jaguar feeding ecology investigation: the direct method of GPS telemetry location clusters with results from the indirect method of scat analysis. Our main objective was to evaluate each method, and we hypothesize that the scat analysis—cheaper and less invasive—is efficient in describing the feeding habits of jaguars. Our investigations were particularly focused on assessing whether the prey communities detected by each method are quantitatively similar and whether both methods show the same pattern of occurrence for the main prey in the jaguar diet.

## Methods

### Study area

The study was conducted at a private, 460 km^2^ cattle ranch (approximately 6,000 head of cattle) in southern Pantanal (19°57'S, 56°25'W). The area is characterized by a mosaic of natural vegetation comprised mainly by grassland, cerrado woodland (cerradão), cerrado (bush savanna), marshes, semideciduous forest, gallery forest, and floating vegetation [43]. A hot and wet season extends from October to March, when the region’s rivers flood a large portion of the area. A warm and dry season extends from April to September.

### GPS location clusters

GPS location clusters were obtained from radio collars. Jaguars were captured with the aid of trained hounds and immobilized with tiletamine hydrochloride and zolazepam hydrochloride (Telazol^®^ 6–10mg/kg). The sedative was administered intramuscularly using a dart fired from a CO2 pistol or a rifle. Upon darting the animal, the hounds were removed from the immediate area for safety [44]. We examined each jaguar for body condition, sex, age and weight, and then fitted them with a GPS collar (Simplex, Televilt International, Sweden). After handling we released them at the site of capture, monitoring from distance until they were able to leave the site on their on.

Capture and handling protocols were approved by the Brazilian Institute of Environment and Natural Resources (permit B-23-9114). All procedures were accompanied by an experienced veterinary. All efforts were made to minimize distress to both dogs and jaguars, and no animals or wildlife were harmed in the course of the study.

The GPS collars were scheduled to obtain seven locations/night in 2002 (fixes every 2 h between 1800 and 0600 h) and 12 locations/day from the end of 2002 to 2004 (fixes every 2 h through 24-h period). With the aid of an aircraft, these data were retrieved remotely by a radio receiver (RX-900; Televilt International) at 21-day intervals, and were plotted on a map of the study area (1:100,000) using ArcView (Environmental Systems Research Institute, Inc., Redlands, California). We classified all clusters of consecutive locations within a 100-m radius as a potential kill site [39]. Inserting the coordinates of one or more locations of those clusters into a portable GPS unit, the research team then explored the area for prey remains over a maximum distance of 100 m in diameter. Additional information on the methods described above can be found in [15].

### Scats collection, handling and prey identification

We collected scat samples opportunistically during fieldwork. We selected only the samples collected during the period when the jaguars wore the GPS collars (November 2001 –April 2004). We distinguished jaguar scats from those of puma (*Puma concolor*) based on their general appearance (only the ones with a diameter > 4 cm) as well as their association with nearby footprints and the exact locations of monitored jaguars. We are aware of the importance of genetic markers for confirming the predators identity (e.g. [35,45]). However, by the time we collected the scats samples, the field of molecular analysis of scats was in its early days and our samples were not properly stored for this kind of procedure. As a security measure, we choose to discard samples we could not rely that were from jaguars.

Collected samples were dehydrated in a screen box exposed to the sun and stored in paper bags. In the laboratory, we put the samples in two layers of pantyhose handmade bags and washed them in a semi-automatic washing machine (Atlanta, Newmaq^®^) for two or three cycles.

To test for hair contamination between samples washed together, we performed an experimental trial. We used scats that we discarded either due to lack of information or because they belonged to other species. For the experimental trial, we washed between two and six samples together with some “fake samples” made of cotton and small stones to simulate the weight of real scat samples. We validated our procedure after noting that no hair entered the fake samples.

Food items found in scats were identified taxonomically through the examination of hair, hair microstructure patterns [46], teeth, claws, nails, osteoderms, scales and feathers. We considered each prey found in a scat sample as an independent capture or one individual. In order to minimize pseudo-replication, subsequent samples collected in close proximity on the same day and containing the same prey species were discarded (personal observation, [27]). We described the components present in the scats in terms of relative frequency of occurrence (number of times a prey species was found relative to total prey).

### Data analysis

To compare the frequency of occurrence of food items (prey) to the prey communities found by the two methods, we used the Bray-Curtis similarity index [47–49]. This index is indicated to reflect accurate quantitative similarity between communities [50]. The Bray-Curtis similarity coefficient, *S*, between the two prey communities identified by two different methods is defined as

$$S=100\left(1-\frac{\sum^{\text{​}}\left|y_{i1}-y_{i2}\right|}{\sum_{i}y_{i1}+\sum_{i}y_{i2}}\right)$$

Where *y*_*i1*_ is the amount *i* of one species of prey in GPS clusters and *y*_*i2*_ is the amount *i* of one species of prey in scat analysis. Equivalent frequencies of prey between methods represent a similarity coefficient of 1.

The Pantanal natural climatic variation is known to determine ecological patterns and strongly affect terrestrial organisms [51]. For this reason, we opted to separate the data into two datasets: rainy season (October–March) and dry season (April–September).

Our hypothesis that scats analysis is able to provide a valid description of jaguar feeding habits when compared with GPS location clusters were tested by chi-square tests of independence [52]. We also examined the over time variation on the proportion of species found both in kills from GPS clusters and in scat samples. All the analyses were implemented with the Package MASS [53] on R [54].

## Results

Ten jaguars were captured and monitored with GPS collars (5 adult males, 1 subadult male, and 4 adult females) [15]. We found a total of 431 kill at GPS location clusters and, concurrently, we identified 153 prey items in 125 jaguar scat samples. The species found through both methods are presented in Table 1.

**Table 1:** Jaguar prey species identified through two main methods, kills found at GPS location clusters of 10 radio-collared jaguars, and prey remains found in 125 scats. November 2001 to April 2004, southern Pantanal, Brazil.

		Kills		Scats	
		n = 431		n = 153	
Prey species		Total	%	Total	%
Cattle	*Bos taurus*	135	31.32	55	35.95
Caiman	*Caiman yacare*	107	24.83	24	15.69
Peccaries*	*Tayassu pecari/Pecari tajacu*	93	21.58	30	19.61
Feral hog	*Sus scrofa*	17	3.94	1	0.65
Marsh deer	*Blastocerus dichotomus*	16	3.71	1	0.65
Giant anteater	*Myrmecophaga tridactyla*	13	3.02	2	1.31
Capybara	*Hydrochoerus hydrochaeris*	9	2.09	5	3.27
Lesser anteater	*Tamandua tetradactyla*	7	1.62	7	4.58
Armadillos*	*Dasypus novemcinctus/Euphractus sexcinctus*	6	1.39	1	0.65
Deer*	*Mazama*sp./*Ozotoceros bezoarticus*	6	1.39	6	3.92
Coati	*Nasua nasua*	5	1.16	5	3.27
Maned wolf	*Chrysocyon brachyurus*	3	0.70	-	-
Crab-eating fox	*Cerdocyon thous*	3	0.70	-	-
Raccoon	*Procyon cancrivorus*	3	0.70	3	1.96
Tapir	*Tapirus terrestris*	2	0.46	-	-
Capuchin monkey	*Sapajus libidinosus*	-	-	1	0.65
Felid	ni	-	-	1	0.65
Agouti	*Dasyprocta azarae*	-	-	1	0.65
Tapeti	*Sylvilagus brasiliensis*	-	-	1	0.65
Gray four-eyed opossum	*Philander opossum*	-	-	1	0.65
Small rodent	ni	-	-	1	-
Jabiru stork	*Jabiru mycteria*	1	0.23	0	-
Boat-billed heron	*Cochlearius cochlearius*	1	0.23	0	-
Great egret	*Ardea alba*	1	0.23	0	-
Bird	ni	0	0	5	3.27
Red-footed tortoise	*Chelonoidis carbonaria*	1	0.23	0	-
Anaconda	*Eunectes* sp.	1	0.23	0	-
Caiman lizard	*Dracaena paraguayensis*	1	0.23	0	-
Lizard	ni	0	0	1	0.65
Crab	ni	-	-	1	
		431		153	

*More than one species combined.

ni = unidentified species.

The similarity of the total prey occurrence between the two methods was high (*S* = 77.34%). The three main prey detected by both methods were cattle (*Bos taurus*), caiman (*Caiman yacare*) and peccaries (*Tayassu pecari* and *Pecari tajacu*) (Table 1). Together, these species accounted for more than 70% of the jaguar diet according to both methods and in both seasons (Table 2). For that reason, we chose to use the data for these 3 main prey only to compare the efficiency of each method.

**Table 2:** Frequency of occurrence (FO) and relative frequency of occurrence (%O) of kills found on jaguars GPS location clusters, and prey remains found in 125 jaguar scats, during dry and wet seasons from 2001 to 2004, Southern Pantanal, Brazil.

	Kills					Scats			
	Dry season		Wet season			Dry season		Wet season	
	n = 260		n = 171			n = 114		n = 39	
Prey	FO	% O	FO	% O	Prey	FO	% O	FO	% O
Cattle	93	35.77	42	24.56	Cattle	48	42.11	7	17.95
Caiman	54	20.77	53	30.99	Peccary	20	17.54	10	25.64
Peccary	49	18.85	44	25.73	Caiman	16	14.04	8	20.51
Marsh deer	10	3.85	6	3.51	Coati	5	4.39	0	0.00
Tapir	2	0.77	-	-	Lesser anteater	4	3.51	3	7.69
Feral hog	9	3.46	8	4.68	Capybara	3	2.63	2	5.13
Giant anteater	9	3.46	4	2.34	Deer	3	2.63	3	7.69
Capybara	8	3.08	1	0.58	Raccon	3	2.63	-	-
Brocket deer	4	1.54	2	1.17	Giant anteater	2	1.75	-	-
Maned wolf	3	1.15	-	-	Bird	2	1.75	3	7.69
Lesser anteater	5	1.92	2	1.17	Small mammals	2	1.75	-	-
Coati	4	1.54	1	0.58	Marsh deer	1	0.88	-	-
Red-footed tortoise	1	0.38	-	-	Feral hog	1	0.88	-	-
Crab-eating fox	2	0.77	1	0.58	Armadillo	1	0.88	-	-
Raccon	2	0.77	1	0.58	Felid ni	1	0.88	-	-
Armadillo	2	0.77	4	2.34	Lizard ni	1	0.88	-	-
Anaconda	1	0.38	-	-	Crab	1	0.88	-	-
Bird*	1	0.38	2	1.17	Capuchin monkey	-	-	1	2.56
Caiman lizard	1	0.38	-	-	Agouti	-	-	1	2.56
					Tapeti	-	-	1	2.56

* More than one species combined.

ni = unidentified species.

**Fig 1:**
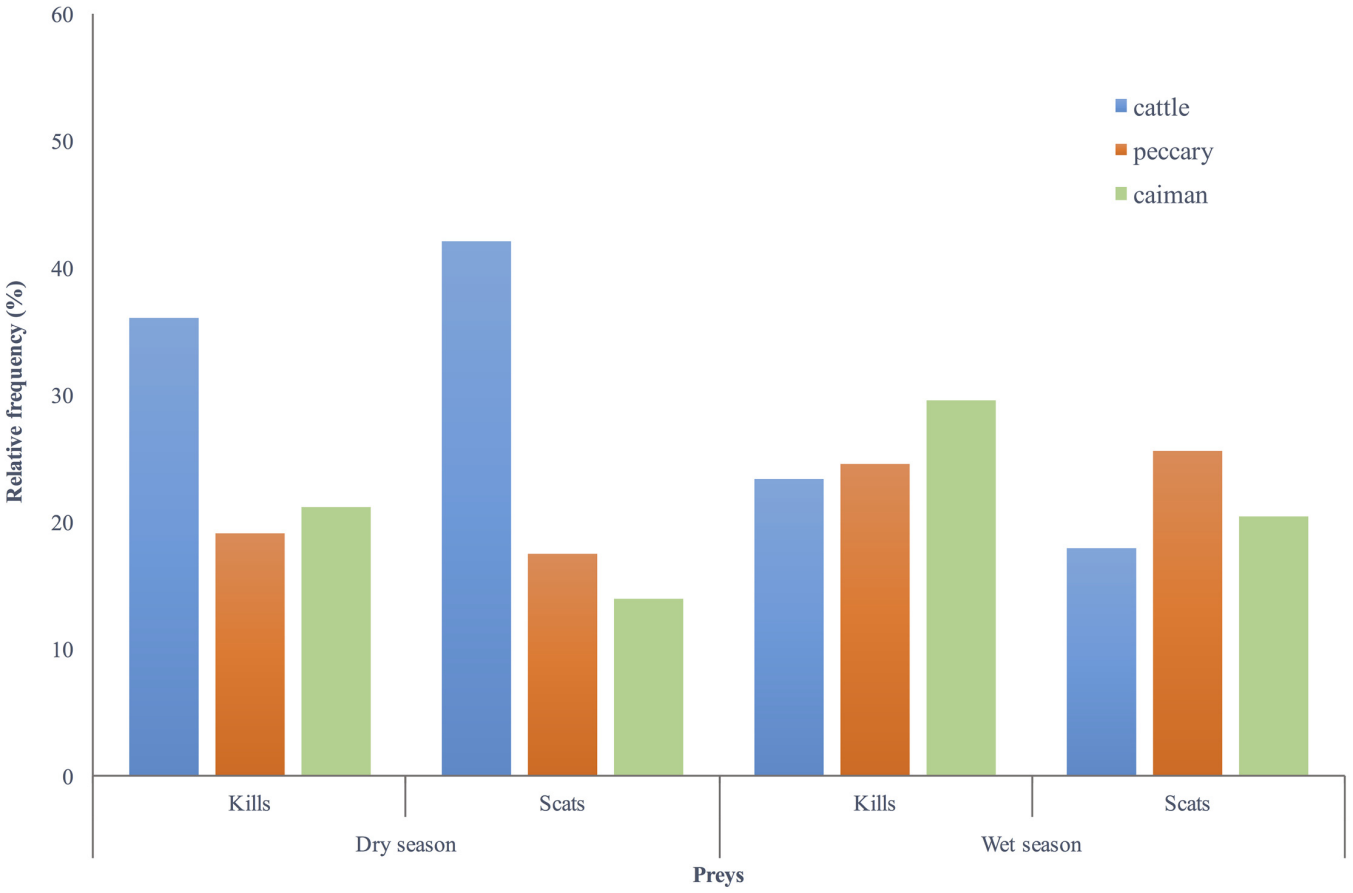
Relative frequency of occurrence of the 3 main prey found in GPS location clusters of 10 radio-collared jaguars (Kills) and in 125 jaguar scats (Scats) during dry and wet seasons from 2001 to 2004. Southern Pantanal, Brazil.

**Fig 2:**
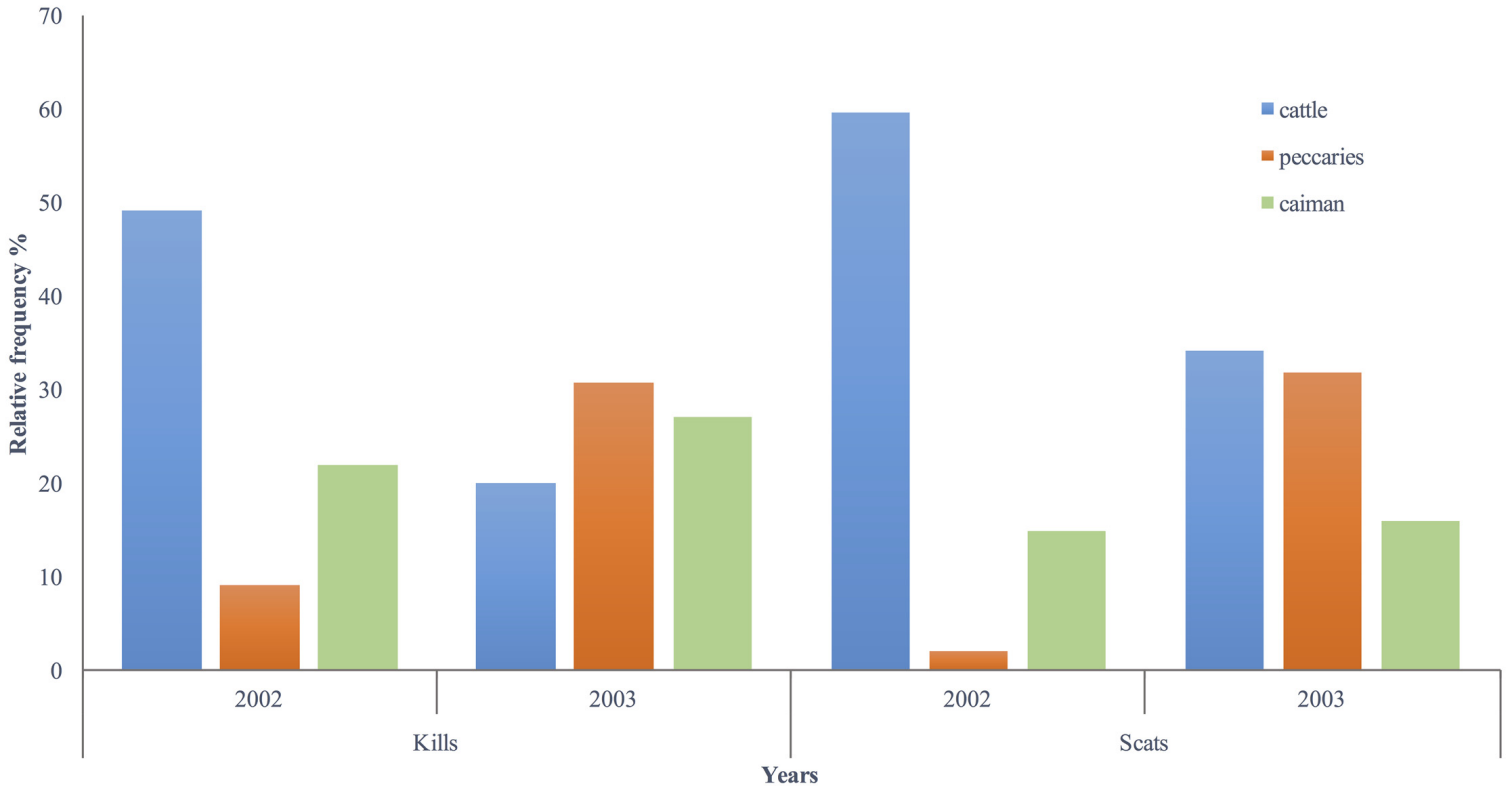
Relative frequency of occurrence of the 3 main prey found in GPS telemetry clusters of 10 radio-collared jaguars (Kills) and in 125 jaguar scats (Scats) during the years of 2001 and 2002. Southern Pantanal, Brazil.

We found no difference in the frequency of the main prey detected between both methods during the dry season (χ^2^_2_ = 2.83, *P* = 0.24) or during the wet season (χ^2^_2_ = 0.69, *P =* 0.70) (Fig 1). The GPS method was able to detect a variation in prey composition as an influence of season (climatic influence of dry season x wet season) (χ^2^_2_ = 10.14, *P* = 0.006), and so did the scat analysis χ^2^_2_ = 6.54, *P* = 0.03) (Fig 1). Both methods were able to record a peak in livestock predation during the year of 2002, followed by a reduction in livestock consumption and an increase in peccary predation in 2003 (Fig 2). The year of 2002 was the driest (550 mm of rainfall) and the year of 2003 was the wettest (1,700 mm of rainfall) of 8 consecutive years (1997–2004) on the study site. The GPS method detected a reduction from 49% of cattle in jaguar kills in 2001 to 20% in 2002 (χ^2^_1_ = 20.48, *P* < 0.001), and scats analysis detected a reduction of 59% to 39% (χ^2^_1_ = 3.93, *P* = 0.04). On the other hand, the proportion of peccaries in jaguar kills increased from 9% in 2002 to 31% in to 2003 (χ^2^_1_ = 24.82, *P* < 0.001), and from 2% to 31% in jaguar scats (χ^2^_1_ = 11.26, *P* < 0.001). Similarly to the GPS method, scat analysis did not find a difference in caiman occurrence between seasons (χ^2^_2_ = 2.66, *P* = 0.10) (Fig 1) or years (Fig 2).

## Discussion

The prey community detected in jaguar diet did not differ when analyzed using either method, GPS location clusters or scat analysis. We found a high similarity between both methods in terms of consumed prey, despite some natural variation due to jaguar individual preference [15].

Our findings differ from previous studies that investigated jaguar diet using both methods. Azevedo and Murray [14] detected capybara as the main prey in jaguar kills (31.6%) and in scats (20,8%). However, the second main prey encountered in scats was deer (red-brocket deer and dwarf red-brocket deer) (19.2%) and these species were not recorded as kills. The data Scognamillo and collaborators [30] collected to investigate jaguar diet also included kills and scats. Their study results indicate a difference on prey proportion among both methods, with livestock as the main jaguar kill (33%) opposed to only 7% of occurrence on scats. However, peccaries accounted for 40% of the jaguar diet based on scats and just 16% based on kills. Jaguar preference for large prey (>15kg) was evident in both methods, as expected for the Pantanal. Prey selection can be related to predator size as carnivores exhibit different feeding strategies according to their body mass [55]. The Pantanal jaguar features a high mean weight, with individuals of >80kg, and have a high energetic demand [15]. Similar to our results, other studies on the Pantanal also showed large prey as the base diet of jaguars, even though the proportion of species was different. Between the years of 1980–1983, Crawshaw and Quigley [56] investigated jaguar kills in the same region of our study. They found the main prey to be cattle, peccaries and capybaras. Azevedo and Murray [14], also in a cattle ranch, found that capybara was the main prey in jaguar diet, followed by cattle, deer and caiman. In Venezuela llanos, male jaguars also weight an average of >80 kg and selected for large prey (capybaras and peccaries) [30].

Conversely, rainforest jaguar show a diet less concentrated on large prey and with a more even composition. Garla and collaborators [27] found 40.5% of jaguar prey base in Brazilian Atlantic Forest to be comprised of medium sized (which they considered 3kg-10kg) species, 27.7% of large sized (>10kg) and 23.4% of small sized species (1-3kg). In Iguaçu National Park, also in the Brazilian Atlantic Forest, 49. 4% of jaguar diet was comprised by medium-sized prey, followed by 46.6% of large-sized prey species [34].

Another explanation for the variation in prey composition in a predator diet is related to prey availability, which can explain the differences between our findings and previous studies. Rabinowitz and Notthingham [19] found that the occurrence of prey in jaguar scats was associated with the availability of prey species in Guam Bank (Belize), showing the opportunist nature of jaguars as predators. Also in Guam Bank, Weckel and colleagues [57] confirmed the previous observations, but noted a tendency to large prey selection.

Some large prey species where detected only by the GPS location cluster method, namely maned wolves, crab-eating foxes and tapirs. Quigley and Crawshaw [56] monitored jaguars with radio-telemetry in the same area of our study and also recorded tapir as a jaguar prey. However, other studies that used only scats to describe jaguar diet did not record tapir, even though its occurrence in the study areas were known (e.g. [19,20,34,57]). Beside the low occurrence of tapirs on jaguar diet, the difficulty in detecting tapirs on scats may be due the small surface to volume ratio of tapirs [35,58]. That associated with the species shortage of hair, can produce scats without identifiable remains [35].

Jaguars tend to remain feeding on the carcasses of large animals for a long time, returning frequently [15]. Monitored individuals remained for an average of 23.3 hours at the carcasses of prey weighing 45–200 kg [15]. Consequently, an individual prey may be associated with several scat samples, and we expected the method of scat collection to overestimate predation on large prey. However, we did not observe that overestimation in our study. It is important to emphasize that we took care to avoid the collection of multiple scats from a single jaguar that fed on a single prey over several meals. When several scats were found in close proximity during a single occasion, and contained the same food item, we selected only one sample for the analysis, thereby minimizing the risk of bias due to pseudo-replication of large prey.

We also assessed whether the GPS location cluster method was biased towards larger prey and missed detection of smaller prey [15,59,60]. As opposed to our expectations, the direct method of locating kills (GPS) enabled us to encounters maller prey species as well. Some medium-sized species such as red-footed tortoise (*Chelonoidis carbonaria*) and armadillos (*Dasypus novencinctus* and *Euphractus sexcinctus*) were located due to their carapace, which is not eaten by the jaguar. The direct method enabled us to record also two small prey: a boat-billed heron (*Cochlearius cochlearius*) and an egret (*Ardea alba*). However, small prey found in scats (but not in GPS clusters) represented less than 5% of the diet. Missing smaller prey may be more problematic in areas where they are more representative of jaguar diet, as in forested areas. Some small prey found in scats, such as crab and small rodent, are probably consumed during rare encounters between jaguars and vulnerable animals, or they are consumed indirectly as the stomach contents of larger prey [20].

Additionally, given the comparable results in terms of species occurrence in the diet, we were able to detect similar patterns of variation in the frequency of main prey consumed in wet and dry seasons, as well as among the years of 2002 and 2003. The remarkable patterns revealed by both methods show a peak of predation on livestock in dry seasons and in the driest year (2002) and an increase in the consumption of peccaries in the wettest year (2003). This peak in livestock consumption may be a reflection of an increase in livestock availability during the dry season, as the cattle herds can be found spread in large portions of the ranch. In addition, the dry season also coincides with the livestock calving season, which increases the availability of calves. A dominance of calves in jaguar kills was observed, as from the 135 cattle records in GPS clusters, 94 (69.63%) were of calves. Furthermore, during the dry season, natural prey move toward the remaining bodies of water, while cattle remains confined within fenced pastures. In contrast, during the wet season, large portions of the study area remain flooded and cattle herds group together at higher ground pastures, while native prey like caiman can be found widely dispersed through the landscape and available to more jaguars [15]. Indeed, the proportion of caiman in jaguar kills and scats increased during the wet season.

The increasing consumption of peccaries coincided with a decrease in predation of domestic livestock, which suggests the importance of maintaining native prey species in order to minimize jaguar-human conflicts that result from predation on livestock [26]. Important jaguar prey such as peccaries can be associated with forested areas, where most fruit are produced [51]. The conversion of forested landscapes into grasslands, for cattle grazing, can severely threaten vertebrate communities and trophic processes [51].

Despite the large number of studies that used scat analysis to examine jaguar diet, to our knowledge none focused on evaluating the efficiency of this method. Together, the direct and indirect methods we present here allowed the construction of a comprehensive diet database, with about 33 recorded taxa. Thus, our results also indicate the importance of joint and complementary studies for dietary descriptions. Given there are concerns over perceived versus real threats jaguars pose to livestock producers, data on the feeding habits of the species are important to subsidize jaguar conservation actions. Furthermore, considering the conflict caused by livestock predation, an accurate estimation of jaguar feeding habits is essential for the long-term conservation of the species.

## References

[1] Fisher SM, Russ GR, Alcala AC, Ecol M, Ser P, Sci BM, et al Ecological Meltdown in Predator-Free Forest Fragments. Science. 2001;294: 1923–6. 10.1126/science.1064397 11729317

[2] Ryall KL, Fahrig L. Response of predators to loss and fragmentation of prey habitat: A review of theory. Ecology. 2006;87: 1086–1093. 10.1890/0012-9658(2006)87[1086:ROPTLA]2.0.CO;2 16761585

[3] Roemer GW, Gompper ME, Van Valkenburgh B. The Ecological Role of the Mammalian Mesocarnivore. Bioscience. 2009;59: 165–173. 10.1525/bio.2009.59.2.9

[4] Estes J a, Terborgh J, Brashares JS, Power ME, Berger J, Bond WJ, et al Trophic downgrading of planet Earth. Science. 2011;333: 301–306. 10.1126/science.1205106 21764740

[5] Ripple WJ, Estes J a, Beschta RL, Wilmers CC, Ritchie EG, Hebblewhite M, et al Status and ecological effects of the world’s largest carnivores. Science (80-). 2014;343: 1241484 10.1126/science.1241484 24408439

[6] Wallach AD, Izhaki I, Toms JD, Ripple WJ, Shanas U. What is an apex predator? Oikos. 2015;124: 1453–1461. 10.1111/oik.01977

[7] Caso A, López-González C a., Payan E, Eizirik E, Oliveira TG, Leite MRP, et al Panthera onca [Internet]. The IUCN Red List of Threatened Species 2008. 2008 Available: 10.2305/IUCN.UK.2008.RLTS.T15953A5327466.en

[8] Sanderson EW, Redford KH, Chetkiewicz CLB, Medellin RA, Rabinowitz AR, Robinson JG, et al Planning to save a species: The jaguar as a model. Conserv Biol. 2002;16: 58–72. 10.1046/j.1523-1739.2002.00352.x 35701976

[9] Hoogesteijn R. Strategies for reducing conflicts between jaguars and cattle. Wild Felid Monit. 2010;3: 1–32.

[10] Loveridge AJ, Wang SW, Frank LG, Seidensticker J. People and wild felids: conservation of cats and management of conflicts In: Macdonald DW, Loveridge AJ, editors. The Biology and Conservation of Wild Felids. Oxford University Press; 2010 pp. 161–195.

[11] Polisar J, Maxit I, Scognamillo D, Farrell L, Sunquist ME, Eisenberg JF. Jaguars, pumas, their prey base, and cattle ranching: Ecological interpretations of a management problem. Biol Conserv. 2003;109: 297–310. 10.1016/S0006-3207(02)00157-X

[12] Azevedo FCC, Murray DL. Evaluation of potential factors predisposing livestock to predation by jaguars. J Wildl Manage. 2007;71: 2379 10.2193/2006-520

[13] Quigley HB. Ecology and conservation of the jaguar in the Pantanal region, Mato Grosso do Brazil. 1987.

[14] Azevedo FCC, Murray DL. Spatial organization and food habits of jaguars (Panthera onca) in a floodplain forest. Biol Conserv. 2007;137: 391–402. 10.1016/j.biocon.2007.02.022

[15] Cavalcanti SMC, Gese EM. Kill rates and predation patterns of jaguars (Panthera onca) in the southern Pantanal, Brazil. J Mammal. 2010;91: 722–736. 10.1644/09-MAMM-A-171.1

[16] Quigley HB, Crawshaw PG. A conservation plan for the jaguar in the Pantanal region of Brazil. Biol Conserv. 2000;61: 149–157. 10.1016/0006-3207(92)91111-5

[17] Schaller GB, Vasconcelos JMC. Jaguar predation on capybara. Z Saeugetierk. 1978;43: 296–301.

[18] Schaller GB, Crawshaw PGJ. Movement Patterns of Jaguar Biotropica. Cambridge: Cambridge University Press; 1980;12: 161–168.

[19] Rabinowitz AR, Nottingham BG Jr.. Ecology and behaviour of the Jaguar (Panthera onca) in Belize, Central America. J Zool. 1986;210: 149–159. Available: http://onlinelibrary.wiley.com/doi/10.1111/j.1469-7998.1986.tb03627.x/abstract

[20] Emmons LH. Comparative feeding ecology of felids in a neotropical rainforest. Behav Ecol Sociobiol. 1987;20: 271–283. 10.1007/BF00292180

[21] Crawshaw PG. Comparative Ecology of Ocelot (*Felis pardalis*) and Jaguar (*Panthera onca*) in a Protected Subtropical Forest in Brazil and Argentina. University of Florida 1995.

[22] Aranda M, Sánchez-Cordero V. Prey Spectra of Jaguar (Panthera onca) and Puma (Puma concolor) in Tropical Forests of Mexico. Stud Neotrop Fauna Environ. 1996;31: 65–67. 10.1076/snfe.31.2.65.13334

[23] Facure KG, Giaretta A. Food habits of carnivores in a coastal Atlantic forest of southeastern Brazil. Mammalia. 1996;60: 499–502.

[24] Chinchilla FA. Diets of Panthera onca, Felis concolor and Felis pardalis (Carnivora: Felidae) in Parque Nacional Corcovado, Costa Rica. Rev Biol Trop. 1997;45: 1223–1229.

[25] Taber AB, Novaro AJ, Neris N, Colman FH. The Food Habits of Sympatric Jaguar and Puma in the Paraguayan Chaco. Biotropica. 1997;29: 204–213. 10.1111/j.1744-7429.1997.tb00025.x

[26] Núñez R, Miller B, Lindzey F. Food habits of jaguars and pumas in Jalisco, Mexico. J Zool. 2000;252: 373–379. 10.1111/j.1469-7998.2000.tb00632.x

[27] Garla RC, Setz EZF, Gobbi N. Jaguar (Panthera onca) Food Habits in Atlantic Rain Forest of Southeastern Brazil1. Biotropica. 2001;33: 691–696. 10.1111/j.1744-7429.2001.tb00226.x

[28] Leite Y, Galvão F. El jaguar, el puma y el hombre en tres áreas protegidas del bosque atlántico costero de Paraná, Brasil In: Medellin RA, Equihua C, Chetkiewicz CLB, Crawshaw PG, Rabinowitz AR, Redford KH, et al, editors. El Jaguar en el Nuevo Milenio. 2002.

[29] Dalponte J. Dieta del jaguar y depredacion de ganado en el norte de Pantanal In: Medellin RA, Equihua C, Chetkiewicz C-LB, Crawshaw PG, Rabinowitz AR, Redford KH, editors. El jaguar en el nuevo milenio. Universidad Nacional Autonoma do Mexico/Wildlife Conservation SOciety; 2002.

[30] Scognamillo D, Maxit IE, Sunquist M, Polisar J. Coexistence of jaguar (Panthera onca) and puma (Puma concolor) in a mosaic landscape in the Venezuelan llanos. J Zool. 2003;259: 269–279. 10.1017/S0952836902003230

[31] Novack AJ, Main MB, Sunquist ME, Labisky RF. Foraging ecology of jaguar (Panthera onca) and puma (Puma concolor) in hunted and non-hunted sites within the Maya Biosphere Reserve, Guatemala. J Zool. 2005;267: 167–178. 10.1017/S0952836905007338

[32] Ceballos G, Chávez C, Zarza H, Manterola C. Ecología y conservación del jaguar en la región de calakmul. CONABIO Biodiversitas. 2005;62: 1–7.

[33] Rosas-Rosas OC, Bender LC, Valdez R. Jaguar and Puma Predation on Cattle Calves in Northeastern Sonora, Mexico. Rangel Ecol Manag. 2008;61: 554–560. 10.2111/08-038.1

[34] Azevedo FCC. Food Habits and Livestock Depredation of Sympatric Jaguars and Pumas in the lguagu National Park Area, South Brazil. Biotropica. 2008;40: 494–500. Available: http://onlinelibrary.wiley.com/doi/10.1111/j.1744-7429.2008.00404.x/full/npapers3://publication/uuid/7CFC4FD8-E402-471D-A05D-3C6A90767F71

[35] Foster RJ, Harmsen BJ, Valdes B, Pomilla C, Doncaster CP. Food habits of sympatric jaguars and pumas across a gradient of human disturbance. J Zool. 2010;280: 309–318. 10.1111/j.1469-7998.2009.00663.x

[36] Sollmann R, Betsch J, Furtado MM, Hofer H, Jácomo AT a, Palomares F, et al Note on the diet of the jaguar in central Brazil. Eur J Wildl Res. 2013;59: 445–448. 10.1007/s10344-013-0708-9

[37] Hernández-SaintMartín AD, Rosas-Rosas OC, Palacio-Núñez J, Tarango-Arambula L a., Clemente-Sánchez F, Hoogesteijn AL. Food Habits of Jaguar and Puma in a Protected Area and Adjacent Fragmented Landscape of Northeastern Mexico. Nat Areas J. 2015;35: 308–317. 10.3375/043.035.0213

[38] Gómez-Ortiz Y, Monroy-Vilchis O, Mendoza-Martínez GD. Feeding interactions in an assemblage of terrestrial carnivores in central Mexico. Zool Stud. 2015;54: 16 10.1186/s40555-014-0102-7 PMC6661297 31966103

[39] Anderson CR, Lindzey FG. Estimating cougar predation rates from GPS location clusters. J Wildl Manage. 2003;67: 307–316.

[40] Bacon MM, Becic GM, Epp MT, Boyce MS. Do GPS clusters really work? Carnivore diet from scat analysis and GPS telemetry methods. Wildl Soc Bull. 2011;35: 409–415. 10.1002/wsb.85

[41] Pitman RT, Swanepoel LH, Ramsay PM. Predictive modelling of leopard predation using contextual Global Positioning System cluster analysis. J Zool. 2012;288: 222–230. 10.1111/j.1469-7998.2012.00945.x

[42] Knopff KH, Knopff AA, Warren MB, Boyce MS. Evaluating Global Positioning System Telemetry Techniques for Estimating Cougar Predation Parameters. J Wildl Manage. 2009;73: 586–597. 10.2193/2008-294

[43] Harris MB, Tomas W, Mourão G, Da Silva CJ, Guimarães E, Sonoda F, et al Safeguarding the pantanal wetlands: Threats and conservation initiatives. Conserv Biol. 2005;19: 714–720. 10.1111/j.1523-1739.2005.00708.x

[44] Morato RG, Moura CA, Crawshaw PG. Chemical restraint of free ranging jaguars (Panthera onca) with tiletamine-zolepezam combination. El jaguar en el nuevo milenio. 2002 pp. 91–99.

[45] Farrell LE, Roman J, Sunquist ME. Dietary separation of sympatric carnivores identified by molecular analysis of scats. Mol Ecol. 2000;9: 1583–1590. 10.1046/j.1365-294X.2000.01037.x 11050553

[46] Quadros J, Monteiro-Filho ELD a. Revisão conceitual, padrões microestruturais e proposta nomenclatória para os pêlos-guarda de mamíferos brasileiros. Revista Brasileira de Zoologia. 2006 pp. 279–292. 10.1590/S0101-81752006000100023

[47] Bray JR, Curtis JT. An Ordination of the upland forest community of southern Wisconsin.pdf. Ecology Monographs. 1957 pp. 325–349. 10.2307/1942268

[48] Legendre P, Legendre L. Numerical ecology Second english edition Numerical Ecology Second English Edition. 2003.

[49] Clarke KR, Somerfield PJ, Chapman MG. On resemblance measures for ecological studies, including taxonomic dissimilarities and a zero-adjusted Bray-Curtis coefficient for denuded assemblages. J Exp Mar Bio Ecol. 2006;330: 55–80. 10.1016/j.jembe.2005.12.017

[50] Bloom SA. Similarity Indices in Community Studies: Potential Pitfalls. Marine Ecology. 1981 pp. 125–128. 10.3354/meps005125

[51] Desbiez ALJ, Bodmer RE, Tomas WM. Mammalian Densities in a Neotropical Wetland Subject to Extreme Climatic Events. Biotropica. 2010;42: 372–378. 10.1111/j.1744-7429.2009.00601.x

[52] Zar JH. Biostatistical analysis. 3rd ed. 1996.

[53] Venables WN, Ripley BD. Modern Applied Statistics with S. Issues of Accuracy and Scale. 2002; 868 10.1198/tech.2003.s33

[54] R Core Team. R: A Language and Environment for Statistical Computing [Internet]. R Foundation for Statistical Computing 2014 Available: http://www.r-project.org/

[55] Carbone C, Teacher A, Rowcliffe JM. The costs of carnivory. PLoS Biol. 2007;5: 0363–0368. 10.1371/journal.pbio.0050022 PMC1769424 17227145

[56] Crawshaw PG, Quigley HB. Habitos alimentarios del jaguar y el puma en Brasil implicaciones para conservación. El Jaguar en el Nuevo Milenio. 2002 pp. 223–235.

[57] Weckel M, Giuliano W, Silver S. Jaguar (Panthera onca) feeding ecology: Distribution of predator and prey through time and space. J Zool. 2006;270: 25–30. 10.1111/j.1469-7998.2006.00106.x

[58] Wachter B, Blanc AS, Melzheimer J, Höner OP, Jago M, Hofer H. An advanced method to assess the diet of free-ranging large carnivores based on scats. PLoS One. 2012;7: e38066 10.1371/journal.pone.0038066 22715373 PMC3371055

[59] Martins Q, Horsnell WGC, Titus W, Rautenbach T, Harris S. Diet determination of the Cape Mountain leopards using global positioning system location clusters and scat analysis. J Zool. 2011;283: 81–87. 10.1111/j.1469-7998.2010.00757.x

[60] Tambling CJ, Laurence SD, Bellan SE, Cameron EZ, Du Toit JT, Getz WM. Estimating carnivoran diets using a combination of carcass observations and scats from GPS clusters. J Zool. 2012;286: 102–109. 10.1111/j.1469-7998.2011.00856.x PMC3293475 22408290

